# Feasibility of using combined EMG and kinematic signals for prosthesis control: A simulation study using a virtual reality environment

**DOI:** 10.1016/j.jelekin.2015.06.010

**Published:** 2016-08

**Authors:** Dimitra Blana, Theocharis Kyriacou, Joris M. Lambrecht, Edward K. Chadwick

**Affiliations:** a Institute for Science and Technology in Medicine, Keele University, UK; b School of Computing and Mathematics, Keele University, UK; c Biomedical Engineering Department, Case Western Reserve University, USA

**Keywords:** Amputation, Prosthesis, Myoelectric, Transhumeral, Electromyography, Artificial neural network, Control

## Abstract

Transhumeral amputation has a significant effect on a person’s independence and quality of life. Myoelectric prostheses have the potential to restore upper limb function, however their use is currently limited due to lack of intuitive and natural control of multiple degrees of freedom. The goal of this study was to evaluate a novel transhumeral prosthesis controller that uses a combination of kinematic and electromyographic (EMG) signals recorded from the person’s proximal humerus. Specifically, we trained a time-delayed artificial neural network to predict elbow flexion/extension and forearm pronation/supination from six proximal EMG signals, and humeral angular velocity and linear acceleration. We evaluated this scheme with ten able-bodied subjects offline, as well as in a target-reaching task presented in an immersive virtual reality environment. The offline training had a target of 4° for flexion/extension and 8° for pronation/supination, which it easily exceeded (2.7° and 5.5° respectively). During online testing, all subjects completed the target-reaching task with path efficiency of 78% and minimal overshoot (1.5%). Thus, combining kinematic and muscle activity signals from the proximal humerus can provide adequate prosthesis control, and testing in a virtual reality environment can provide meaningful data on controller performance.

## 1 Introduction

There are approximately 5–6000 major limb amputations carried out each year in the UK [12]. Although the proportion of amputees referred with upper limb amputations is only about 5%, they are a population with high functional demands. Trauma is the major reason for upper limb amputation, and this is reflected in the age group affected by the condition, with 66% aged less than 55 years [12]. As a result, loss of the upper limb can have a significant effect on the ability to work, independence, and overall quality of life. Amputees who choose to fit a prosthetic limb onto the remaining arm have two options: a passive (cosmetic) prosthesis, which offers little functional benefit, or an active prosthesis that has the potential to restore upper limb function. Active prostheses are either body-powered, which are controlled by upper body movements through straps and cables, or myoelectric, which are electrically powered and use the residual neuromuscular system for control.

Myoelectric prosthetic designs are continuously improving, with several manufacturers recently introducing devices that include dexterous prosthetic hands with multiple degree of freedom, e.g. i-Limb ultra by Touch Bionics and bebionic from RSLSteeper [16]. However, the development of functional and intuitive control schemes for these devices has not kept pace with the advancement of the hardware. Commercially available myoelectric prostheses are controlled by recording electrical signals (“electromyographic signals” or EMG) generated by the contractions of residual muscles. They use simple control schemes, where EMG from a pair of opposing muscles are used to actuate a single prosthesis motor, with a “mode switch” to transition from one function to the next [11]. This sequential control method can be very slow and unnatural compared to able-bodied upper limb control.

Adoption and use of advanced myoelectric prostheses is currently limited; in the survey conducted by Biddiss and Chau [3], 20% of participants had abandoned prosthesis use, stating aspects of prosthesis design such as limited function and ease of control as important factors in abandonment. To improve functional gain, it is necessary to develop advanced control algorithms and signal processing techniques that would allow the use of more EMG signals to control multiple functions simultaneously. In Pulliam et al. [13], EMG from seven proximal muscles was used to predict the movement of the elbow and forearm, with promising results. Control could be further improved with measurements of residual body motions using accelerometers and other sensors, as shown in Fougner et al. [7], where EMG was combined with accelerometer data, and Akhtar et al. [1], where EMG was combined with shoulder orientation.

In these studies, performance of the control method was evaluated offline, which demonstrates that there is a relationship between input and output signals that can be exploited for intuitive and natural control. However, good offline performance does not necessarily translate to good performance during use, since errors in the training are inevitable and may be difficult for the user to overcome. It is therefore important to test novel control algorithms with the user in the loop. Cost-effective testing can take place in a virtual reality environment, where the “virtual prosthesis” user can practice with different control schemes, choose the preferred method and train in its use, before an actual prosthesis is fitted [15], [9], [8], [4].

In this study, we developed a transhumeral prosthesis controller that combines EMG and kinematic signals from the proximal humerus to predict the movement of the forearm. We chose to use signals from a single inertial sensor on the humerus, and an array of six EMG electrodes placed around the humerus, so that sensors could be embedded into a prosthesis socket in the practical realisation of the method. We also developed a virtual reality environment that uses a headset to create an immersive experience which is easy to learn and can realistically model prosthesis testing tasks. The specific aims of the project were therefore to test the feasibility of the virtual reality environment for simulating prosthesis control and to evaluate the performance of a novel controller both offline and during online, user-in-the-loop performance.

## 2 Methods

**Fig. 1:**
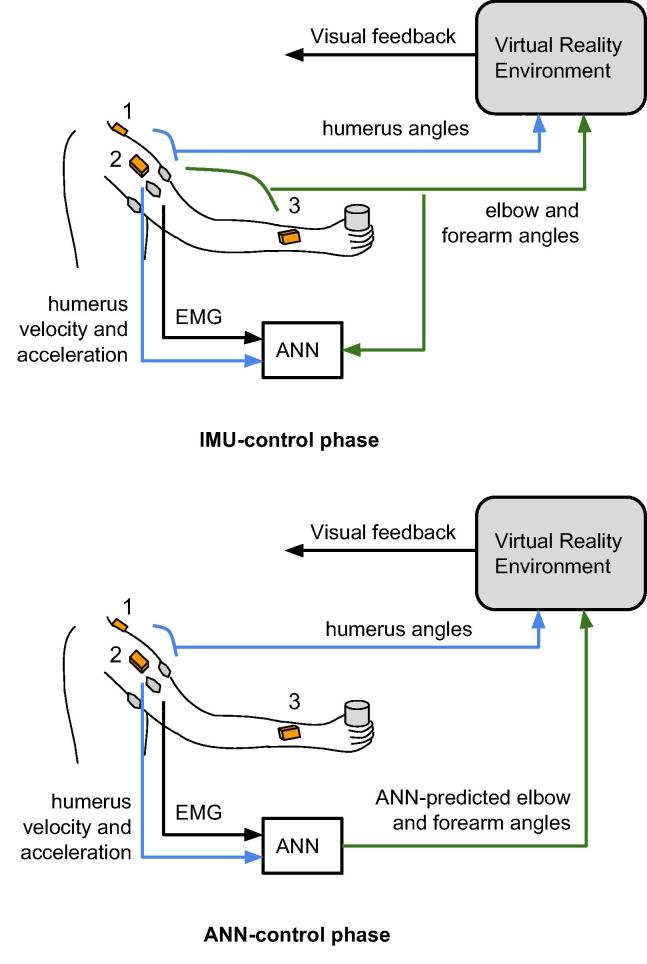
The two experimental phases of the study: the IMU-control and ANN-control phase. Shown are the EMG sensors around the circumference of the humerus (grey ovals), and three IMU (orange boxes, 1: thorax, 2: humerus, 3: forearm). Humeral angles are calculated by the combination of signals from the thorax and humerus IMU, and these are used to control the movement of the virtual humerus in the VRE. Similarly, elbow/forearm angles are calculated by the combination of signals from the humerus and forearm IMU, and these are used in the IMU-control phase to control the movement of the virtual forearm in the VRE. These are also used as output training signals for the ANN, while the input training signals are EMG and humerus angular velocity and linear acceleration, calculated from the humerus IMU. In the ANN-control phase, the ANN outputs are used to control the virtual forearm in the VRE instead of the IMU signals.

Fig. 1 describes the method used in this study. Able-bodied individuals performed reaching movements with their right arms that were translated into movements of a virtual arm in a virtual reality environment. During the movements, EMG and kinematic signals from the humerus were recorded, as well as elbow and forearm angles. These data were used offline to train two time-delayed artificial neural networks (ANN) to predict elbow and forearm angles from processed humerus EMG and kinematic signals. Subsequently, the participants performed similar reaching movements, but the elbow and forearm angles of the virtual arm were now controlled by the trained ANN.

Ten able-bodied subjects with no history of injury to the upper limbs (seven male, three female, age range 22–35 years) participated in the study after giving informed consent; the study was approved by the University Ethics Committee. EMG signals were recorded from six surface electrodes (Biometrics Ltd, UK) placed around the circumference of the humerus, with the first electrode placed on top of the biceps, and the rest at approximately equal spacings. The position along the humerus was chosen as the location of the largest bicep muscle bulk. The EMG signals were amplified, band-pass filtered between 15 and 450 Hz and sampled at 1000 Hz.

Thorax, humerus and forearm movements were recorded using three Xsens MTx inertial measurement units (IMU, Xsens Technologies B.V., the Netherlands). One was placed on the sternum, one on the proximal humerus (distal humerus in two of the subjects with not enough space between the EMG and the top of the humerus) and one on the distal forearm. The humerus and forearm units were placed facing upwards when the humerus was at 90° of flexion and zero rotation, and the forearm at full pronation. We did not consider wrist movement in this study, so the participants kept their wrist at the neutral position with the use of a strap. Data were sampled at 50 Hz from the IMU.

Each IMU comprises a 3D accelerometer, a 3D gyroscope and a 3D magnetometer. Data from the three sensors are combined to measure the 3D orientation of each sensing unit with respect to a global, earth-based frame of reference. Thoracohumeral and elbow angles were calculated using the relative orientation of the three sensing units and a calibration routine to define the anatomical frames of reference of the thorax, humerus and forearm. This calibration routine is described in Cutti et al. [6] and, briefly, consists of the participant being asked to sit with the back straight and the humerus kept alongside the body and performing three five-second trials: (a) a static trial, (b) repeated flexion–extension of the elbow keeping a constant pronation/supination angle (90°), and (c) repeated pronation-supination of the forearm keeping a constant elbow flexion angle (90°).

Thoracohumeral motion was described according to the ISB recommendations as a sequence of three Euler rotations: plane of elevation, elevation angle and axial rotation [18]. These angles were used to translate the movement of the participant’s humerus into the movement of the virtual humerus. Similarly, elbow flexion/extension and forearm pronation/supination angles were used to translate the movement of the participant’s forearm into the movement of the virtual forearm during the first part of the experiment, and provide training data for the ANN. During the second part of the experiment, i.e. the user-in-the-loop testing of the ANN, the elbow and forearm angles measured by the IMU were not used in the virtual environment, but were recorded to quantify the online performance of the ANN. It is important to note that during everyday use of the prosthesis, only the IMU on the humerus would be needed and the calibration described above would not be performed; the multiple IMU and calibration routine are only necessary for testing in the virtual reality environment.

**Fig. 2:**
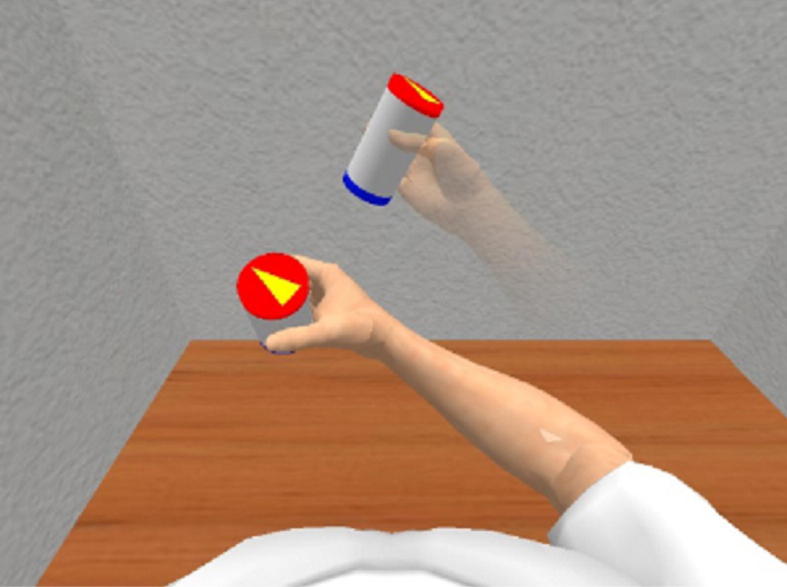
The virtual reality environment, with a first-person view of a virtual person sitting at a desk. The participant controls the arm that is fully opaque, and tries to match the position and orientation of the less opaque (“target”) arm.

The participants were asked to perform reaching movements from a self-selected “initial” position on their lap to various locations in the space in front of them. While performing these movements, they wore a virtual reality headset, the Oculus Rift DVK1 (Oculus VR, Inc., CA, USA), which gave them a first-person view of a virtual person sitting at a desk and tracked their head movements. The virtual reality environment (VRE) was built using GameStudio (Conitec Datasystems, Inc., La Mesa, CA, USA). Targets shown in the VRE directed the reaching movements of the participants. The target was shown as a cylinder held in the hand of a “target arm”, a less opaque arm than the virtual arm controlled by the participant (Fig. 2). The targets were located within a virtual rectangular workspace defined in a coordinate frame originating at the virtual shoulder, with the *x*-axis pointing laterally, the *y*-axis superiorly and the *z*-axis posteriorly. In this frame, the workspace limits were *x*: −10 to 20 cm, *y*: −10 to 20 cm and *z*: −50 to −40 cm. The targets were oriented according to the pronation/supination angle of the target arm, and were either 30° (palm facing upwards) or 90° (neutral) of pronation. The neutral pronation angle was chosen because it allows the performance of activities of daily living such as holding a fork or spoon, which was identified in a survey performed by Atkins et al. [2] as one of the top five activities prosthesis users would like to be able to perform. Similarly, the orientation of the palm facing upwards was chosen because it would allow the prosthesis user to receive small objects such as change during shopping. When the participant moved the virtual arm to within 5 cm of the target location and 30° of the target orientation, the target changed colour to indicate they were within the target, which they then had to maintain for 0.5 s. If they were successful, a new target appeared. The participants were required to return to the initial position and then reach to the new target.

At the start of the experiment, the participants were given the opportunity to familiarise themselves with the virtual environment. They practiced controlling the virtual arm and attaining targets in random locations within the workspace, with a random choice of one of the two orientations, for as long as they required. They were asked to choose a comfortable speed for the movements and keep it roughly the same for the duration of the experiment. The familiarisation took place in trials of 60 s, to avoid fatigue. When they felt ready, they performed a series of 30-s trials to collect training data for the ANN (“IMU-control phase”). For these trials, they were given a predetermined set of 32 target locations, evenly distributed within the workspace, first with 30° and then 90° of target pronation, a total of 64 targets. The kinematic and EMG data recorded during the IMU-control phase were processed every 50 ms to be used as inputs and outputs of the ANN. The kinematic inputs to the ANN were the raw accelerometer and gyroscope data from the IMU placed on the humerus of the participant. The six raw EMG signals were detrended and rectified. An exponential moving average filter was then applied to both kinematic data and processed EMG from the last 150 ms, and the mean was calculated. In the ANN-control phase, the outputs of the ANN were post-processed in a similar way as the inputs for smoothing and increased usability: data from the last 0.5 s were filtered using an exponential moving average filter and averaged.

Two ANN were trained, one for elbow flexion/extension, and one for pronation/supination. They were both two-layer feedforward ANN with a sigmoidal function at the hidden layer and a linear function at the output layer. Three input time delays were used to model the delay between EMG activity and arm movement. Based on the number of inputs and outputs, the number of neurons in the hidden layer was initially chosen to be six. After training, the root mean squared error (RMSE) between the measured angles and ANN-predicted angles was required to be less than 4° for flexion/extension, and 8° for pronation/supination. The accuracy of the flexion/extension angle determines the hand position error, and 4° roughly translates into 2 cm of position error, which can be easily overcome with a small movement of the trunk or humerus. The error tolerance for pronation/supination was doubled because it is generally more difficult to predict the pronation/supination angle from the movement of the humerus and the muscles available following a transhumeral amputation [13]. A small number of neurons in the hidden layer was chosen because a larger number could cause the network to overfit the training data and not generalise well to new data. If the required accuracy could not be achieved with six neurons in the hidden layer, the number would be increased until the prediction was adequate.

After the ANN were trained, the participants were asked to perform ten 30-s trials with randomized targets in different locations than those used for training (“ANN-control phase”). The online prediction errors of the ANN were quantified using the RMSE between the angles measured using the Xsens and the ANN-predicted angles. Additional metrics that describe more detailed performance aspects of the movements in the VRE were calculated, both during the IMU-control and ANN-control phases. These are described in detail in Williams and Krisch [17]:

• Throughput (bits/s) is a measure of the amount of information the participant can convey, and is defined as the index of difficulty of a specific target divided by the movement time required to acquire the target. The index of difficulty is given by: $\mathrm{ID}=\log_{2}(D/W+1)$ where *D* is the distance from the initial position to the target and *W* is the target size (set to 10 cm for all targets in this experiment).
• Overshoot is the measure of the ability of the participant to accurately control the velocity of the virtual arm. It is defined as the number of occurrences of the virtual hand being within the target and then leaving the target before 0.5 s, divided by the total number of targets.
• Path efficiency is a measure of the straightness of the path to the target. It is calculated by dividing the straight-line distance from the initial position to the target by the actual distance travelled by the virtual hand.

The target-reaching task, ANN training and calculation of RMSE and movement metrics were implemented in Matlab (Mathworks, Inc., Natick, MA, USA).

## 3 Results

All participants felt comfortable in the use of the virtual reality environment within about 15 min of familiarisation. For subject 6, the forearm pronation/supination angle could not be measured even following repeated calibration of the IMU, therefore it was not used in the task (i.e. only position, and not orientation was used to determine target acquisition). The data collection for ANN training (IMU-control phase, 64 preset targets) was completed in less than 10 min for all participants (median: 6 min, min: 5 min, max: 11 min).

**Fig. 3:**
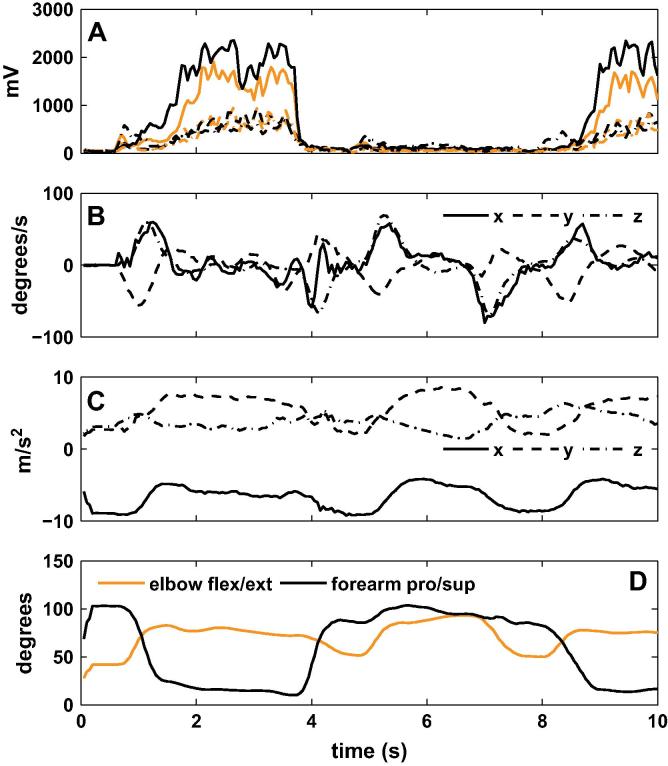
An example of the input (panels A, B and C) and output (panel D) ANN training data. Panel A shows the six processed EMG signals, panel B shows the angular velocity of the humerus IMU, and panel C shows the linear acceleration of the humerus IMU. Panel D shows the elbow flexion/extension and forearm pronation/supination calculated based on the IMU on the humerus and forearm.

Fig. 3 shows an example of the data used to train the ANN. Panel A shows the rectified and filtered EMG data, and panels B and C show the humerus IMU velocity and acceleration data. These 12 signals were the inputs to the ANN, while the outputs were elbow flexion/extension and forearm pronation/supination, calculated from the IMU on the humerus and forearm, shown in panel D. For all participants, six neurons in the hidden layer were sufficient to achieve the required offline accuracy, as shown in Table 1. ANN training with such as small number of neurons was extremely fast (less than 1 min) so the overall training phase required less than 15 min for all participants.

Table 1 also shows the RMS error between the IMU-measured angles and the ANN-predicted angles during the ANN-control phase. As the participants had to use a novel control algorithm, differences in the input signals between training and testing resulted in larger online errors.

**Fig. 4:**
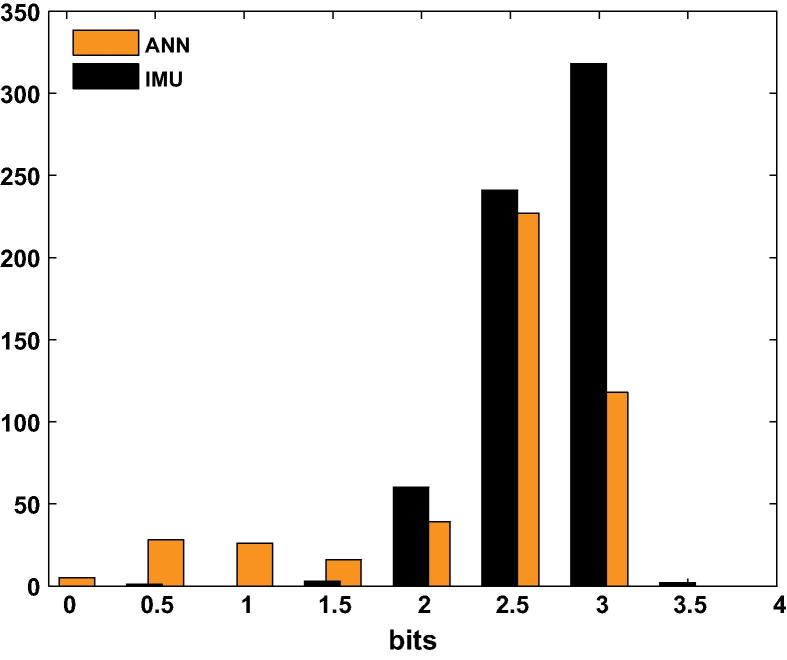
The distribution of the values of the Index of Difficulty, for the targets used during the IMU-control phase (IMU) and the ANN-control phase (ANN).

Fig. 4 shows the distribution of the values of the Index of Difficulty used in the experiment. The target size was fixed, so this depended entirely on the distance to target. The workspace used in the IMU-control phase was slightly larger than the workspace used in the ANN-control phase (so that the ANN did not have to extrapolate during use), thus the histogram for targets seen during training (black bars) includes slightly higher values than the targets seen during testing (median for IMU: 2.76 bits, interquartile range: 2.50–2.94 bits, median for ANN: 2.55 bits, interquartile range: 2.26–2.76 bits).

Table 2 shows the total number of targets hit by each participant during the ten 30-s trials of the ANN-control phase. (The number of targets hit during the IMU-control phase was 64 for all participants.)

**Fig. 5:**
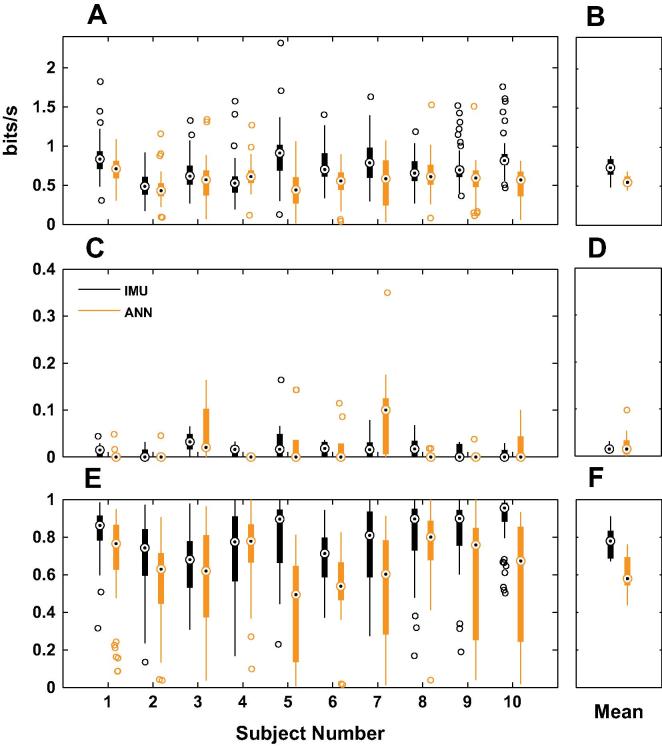
Summary of the movement metrics. Panel A shows the throughput for each subject, in the two experiment phases: IMU-control (IMU, dark bars), and ANN-control phase (ANN, light bars). Panel B shows the mean throughput for each experiment phase. Panel C shows the overshoot per subject, and Panel D shows the mean for each experiment phase. Similarly, Panel E shows the path efficiency per subject, and Panel F shows the mean for each experiment phase.

Fig. 5 summarises the three movement metrics for each subject during the two phases of the experiment: the IMU-control phase (IMU, dark bars), and the ANN-control phase (ANN, light bars). Throughput (panels A and B) was generally low, since it was limited by the values of Index of Difficulty, and the self-selected movement speed (median for IMU: 0.74 bits/s, interquartile range: 0.65–0.84 bits/s, median for ANN: 0.55 bits/s, interquartile range: 0.55–0.62 bits/s, Wilcoxon rank sum test p=0.011). Overshoot (panels C and D) was near zero, suggesting good control of movement speed (median for IMU: 0.015, interquartile range: 0.011–0.019, median for ANN: 0.015, interquartile range: 0.003–0.033, Wilcoxon rank sum test p=0.791). Lastly, when the participants controlled the virtual forearm with the IMU they showed better path efficiency than the ANN controller (panels E and F, median for IMU: 0.78, interquartile range: 0.69–0.83, median for ANN: 0.58, interquartile range: 0.55–0.70, Wilcoxon rank sum test p=0.005).

**Fig. 6:**
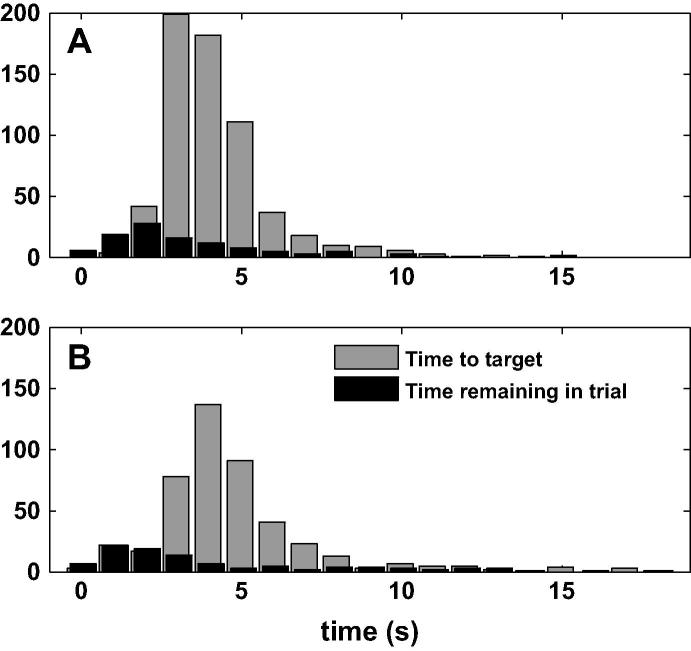
Histogram of the time to target and the time remaining in each trial, after the last target was hit. Panel A shows the IMU-control phase, and Panel B shows the ANN-control phase.

The movement metrics were calculated for the targets acquired, but they do not give any information about targets that were not acquired, e.g. it is possible that during a 30-s trial, the participant reached one target in the first 3 s, and spent the remaining 27 s unsuccessfully reaching for the second target. Consequently, we looked at the time required to reach the target (“time to target” in Fig. 6) and the time remaining in the trial after the last target was acquired (“time remaining in trial” in Fig. 6), to ensure that the time remaining was less than the time to target. In the IMU-control phase (panel A), the median time to target was 3.9 s (interquartile range: 3.2–4.8 s), while the median time remaining in trial was 2.6 s (interquartile range: 1.8–4.7 s). In the ANN-control phase (panel B), the median time to target was 4.3 s (interquartile range: 3.5–5.5 s), while the median time remaining in trial was 2.8 s (interquartile range: 1.4–6.8 s). In both cases, a left-sided Wilcoxon rank sum test showed that the time remaining was less than the time to target (p<0.001).

## 4 Discussion

The results of this study show that a virtual reality environment is a suitable way to evaluate novel prosthesis control algorithms, as participants accommodated to the VRE easily and were able to complete the training tasks within a relatively short amount of time. The immersive environment produced no negative effects in any users and all users were able to perceive the depth aspect of the task and learned to complete the 3D target reaching task.

After a training phase that took less than 15 min for all participants, the offline ANN accuracy exceeded the target of 4° elbow flexion/extension and 8° forearm pronation/supination (mean of 2.7° and 5.5° respectively). Similar results are described in Akhtar et al. [1] (4.1° and 5.4° respectively) when ANN were trained with shoulder orientation and EMG as inputs, but for a single reaching orientation.

Although the offline ANN training errors were within the required tolerance, the online performance showed larger prediction errors (mean of 13.7° and 23.3° respectively). This is to be expected as users have to learn to use a new control algorithm when performing the tasks with the ANN controller as opposed to the training method, and emphasises the importance of evaluating any control algorithm for its user-in-the-loop performance in addition to examining offline fitting errors. However, the online errors are similar to ANN prediction errors in the literature: in Pulliam et al. [13], ANN were trained to predict the elbow flexion/extension and forearm pronation/supination angles using only EMG as input signals, but for a larger variety of movements; the offline training errors were 15.7° and 24.9° respectively.

When controlling the virtual prosthesis with the ANN, users were able to complete the tasks with similar performance metrics to the training phase, although throughput and path efficiency were slightly lower. Scheme et al. [14] used similar movement metrics for the evaluation of an EMG-controlled transradial prosthesis, with a similar range of values for the Index of Difficulty (from 1.59 to 3.46 bits). They compared two control schemes and found throughput values of about 1.1 bits/s, path efficiency of 0.77–0.87 and overshoot of 0.21–0.56. In our study, the participants were not instructed to perform the task quickly, but at a comfortable speed, which resulted in slower movements (reflected in the throughput of 0.55 bits/s) but higher accuracy (overshoot of just 0.015). The path efficiency of 0.78 was similar to the results of Scheme et al. [14].

Although the VRE used in this study allowed investigation of prosthesis controller performance in 3D, there were some limitations. The workspace was quite small, to allow the users to easily see the targets without much head movement; with longer practice in the VRE, users could become more confident in their head movements, and the target space could be expanded to a much larger area. Also, the range of tasks examined was somewhat limited by the lack of interaction with the environment allowed by the VRE. That is, the tasks were limited to goal-directed reaching movements, which although commonly used to assess arm reaching movements, do not represent functional tasks such as activities of daily living. Lambrecht et al. [9] have developed a VRE that simulates prosthesis dynamics and virtual functional assessment tasks such as the Box and Block Test, and this could be used in future experiments. Further development of the VRE could include a haptic robot such as the Haptic Master, to allow users to feel the weight and movements of the prosthesis, and forces from objects in the environment.

Furthermore, the reaching tasks themselves were simplified by the restriction of possible forearm orientations. Since most muscles involved in pronation/supination are not available following a transhumeral amputation, it is not possible to accurately predict a large number of hand orientations, as discussed in Pulliam et al. [13]. The choice of a fully supinated and neutral orientation allowed us to take advantage of the supinating action of the biceps to distinguish the two. Expanding the input sensors to include intramuscular electrodes could allow us to record from the brachialis as well, which flexes the elbow but does not act on the forearm, thus helping the pronation/supination prediction.

In addition to the limitations of the platform, it must be noted that all testing in this study took place with normally-limbed volunteers and not amputees. The participants thus had normal muscle morphology from which to record EMG signals, and it remains to be seen what compromise will be necessary in the case of transhumeral amputees. For this reason, the placement of EMG electrodes in this study was not based on the identification of specific muscles, but electrodes were simply placed fairly uniformly around the proximal humerus. The ANN thus worked with different combinations of muscles in different subjects, providing assurance that it could function with an arbitrary set of inputs, as long as those inputs are repeatable. This gives some confidence that control is feasible even in amputees with abnormal muscle morphology in the proximal humerus.

With regard to the practical realisation of such a system, the neural network used in this study was computationally inexpensive, and it would be simple to implement on an embedded system that could be part of the prosthesis. Following initial training, the network would be able to run fast enough that real-time control with sufficiently low latency would be achievable.

Moreover, for a practically-realisable system, consideration of sensor placement becomes important. The choice of restricting EMG sensors to the proximal humerus, rather than also including shoulder muscles, means that the sensors could all be embedded within the socket of the myoelectric prosthesis. This would also ensure that the EMG signals are fairly repeatable; to account for small changes, a short calibration routine could be developed that adjusts the ANN weights each time the prosthesis is donned.

To assess the feasibility of a self-contained device, we have chosen to use kinematic signals only from the humerus IMU, and not use data from the thorax sensor. However, it should be noted that subjects were seated during the performance of the tasks in this experiment. In a real system, the user may well be walking or moving in some way and this will affect the kinematic signals from the inertial sensors. In order to reject these confounding signals, an additional sensor could be placed somewhere on the trunk such as the anterior shoulder region, and used as a reference. This would, of course, increase the complexity of the system, in particular with regard to donning and doffing the prosthesis.

Work to improve the prediction errors can be focussed on three main areas: user training, neural network structure, and optimisation of sensor placement. The training period for users in this study was short, and it is expected that increasing the training time would lead to a decrease in prediction errors, although care needs to be taken not to fatigue the participants muscles. More advanced network structures such as echo-state networks that are particularly well-suited to modelling time-series data may also bring improvements to prediction error without increasing the computational burden [19]. Finally, positioning of EMG electrodes and inertial sensors was carried out with a focus on convenience. Some optimisation of the location of those sensors (including a move to intra-muscular EMG electrodes) should achieve an improvement in performance, albeit with a cost in terms of simplicity.

Regarding translation of these methods from normally-limbed participants to transhumeral amputees, the question arises of how to collect the training data, where the normal distal limb is not available. In this case, the contralateral limb could be used for training, which was demonstrated in able-bodied participants by Muceli and Farina [10] and was shown to be a feasible method when contralateral kinematic data are available. For bilateral amputees, training could be achieved with teacher imitation, a modality used in Castellini et al. [5], where the amputee imitates the movements of an able-bodied teacher, whose kinematics are used as a target for ANN training. Alternatively, a model-based training approach could be investigated, which involves a musculoskeletal model representing the amputee limb and prosthesis. This could be used to develop an initial controller, that could then be further improved by online adaptation of the ANN.

## 5 Conclusions and further work

We have shown that the use of a virtual reality environment for testing prosthesis control algorithms provides meaningful data on controller performance, and that the combination of kinematic and muscle activity signals from the proximal humerus can be used to provide adequate control of a simulated prosthetic device. Further work will focus on development of the VRE to include object interaction; optimisation of signal processing methods, sensor placement and controller training to reduce prediction errors; and finally expansion of testing and development work to include amputee participants to assess the effects of variable limb morphology on controller performance.

## Conflict of interest

The authors declare that there are no conflicts of interest.

## Figures and Tables

**Table 1:** The offline and user-in-the-loop prediction error of the ANN for each subject. “flex/ext” is elbow flexion/extension, and “pro/sup” is forearm pronation/supination.

Participant	Offline flex/ext (°)	Offline pro/sup (°)	Online flex/ext (°)	Online pro/sup (°)
S1	2.8	7.5	15.8	30.4
S2	2.9	4.8	8.4	16.0
S3	3.4	7.9	11.5	27.1
S4	2.4	5.9	17.6	33.5
S5	2.6	5.9	11.4	19.5
S6	3.0	N/A	20.8	N/A
S7	3.3	7.2	17.1	33.8
S8	2.0	3.4	6.1	13.4
S9	2.1	3.8	9.0	16.1
S10	2.5	3.4	19.4	20.2
mean (std)	2.7 (0.5)	5.5 (1.8)	13.7 (5.1)	23.3 (7.9)

**Table 2:** The total number of targets hit by each participant during the ANN-control phase. This phase consisted of ten 30-s trials for all participants.

Participant	Number of targets hit
S1	62
S2	44
S3	49
S4	53
S5	28
S6	35
S7	40
S8	55
S9	53
S10	40
